# The effect of zinc deficiency and iron overload on endocrine and exocrine pancreatic function in children with transfusion-dependent thalassemia: a cross-sectional study

**DOI:** 10.1186/s12887-021-02940-5

**Published:** 2021-10-22

**Authors:** Suzan O. Mousa, Ebtihal M. Abd Alsamia, Hend M. Moness, Osama G. Mohamed

**Affiliations:** 1 Pediatric Department, Children’s University hospital, Faculty of Medicine, Minia University, El-Minya, Egypt; 2 Department of Clinical Pathology, Minia University hospitals, Minia University, El-Minya, Egypt

**Keywords:** Transfusion-dependent thalassemia, Zinc deficiency, iron overload, Endocrine pancreas, Exocrine pancreas

## Abstract

**Background:**

Children with transfusion-dependent thalassemia (TDT) suffer from secondary hemosiderosis and the delirious effects this iron overload has on their different body organs, including the pancreas. They are also more prone to develop zinc deficiency than the general pediatric population. This study aimed to determine the effect of zinc deficiency and iron overload on the endocrine and exocrine pancreas in TDT children.

**Methods:**

Eighty children, already diagnosed with TDT, were included in this study. We assessed the following in the participant children: serum ferritin, serum zinc, endocrine pancreatic function (oral glucose tolerance test (OGTT), fasting insulin level and from them, HOMA-IR was calculated), and exocrine pancreatic function (serum lipase and serum amylase).

**Results:**

Forty-four TDT children had a subnormal zinc level, while 36 of them had a normal serum zinc level. TDT children with low serum zinc had significantly more impaired endocrine pancreatic function and an abnormally high serum lipase than children with normal serum zinc, *p* < 0.05 in all. Serum zinc was significantly lower in TDT children with serum ferritin above the ferritin threshold (≥2500 ng/ml) than those below (59.1 ± 20.2 vs. 77.5 ± 28.13), *p* = 0.02. TDT children, having a serum ferritin ≥2500 ng/ml, had significantly more frequently impaired endocrine pancreatic function and abnormally high serum lipase than TDT children below the ferritin threshold, *p* < 0.05 in all.

**Conclusion:**

In children with transfusion-dependent thalassemia**,** zinc deficiency aggravates iron-induced pancreatic exocrine and endocrine dysfunction.

## Background

ß-thalassemia is a heterogeneous autosomal recessive hereditary anemia, that is caused by either reduced (ß^+^) or absent (ß^0^) synthesis of the ß-globin chains of the hemoglobin. The reduced or absence of ß-globin chains result in a relative excess of unbound *α*-globin chains, which precipitate in erythroid precursors in the bone marrow leading to their premature death and ineffective erythropoiesis [1].

Transfusion-dependent thalassemia (TDT), previously known as thalassemia major, causes severe anemia with several health problems like enlarged spleen, bone deformities, iron overload, hepatitis infection, and requires regular life-long transfusion therapy and medical supervision [2, 3].

The increased plasma circulating non-transferrin-bound iron (NTBI) species cause iron overload-induced organ dysfunction and is implicated in cellular dysfunction, and cytotoxicity [4]. The unbound iron, together with the chronic hypoxia due to anemia, potentiates iron’s toxic effect on the endocrine glands in patients with thalassemia [5].

Studies have documented endocrine pancreas dysfunction in patients with thalassemia [6, 7], but little has been published about the alterations of the exocrine pancreas in thalassemia.

Chronic blood transfusion in thalassemia changes the micronutrient status [8]. One of the most critical micronutrients deficiencies patients with thalassemia suffer from is zinc, which is an essential trace element in animal and human nutrition and well established in the synthesis of cholesterol, protein, and fats [9]. For multiple reasons, hemoglobinopathies and thalassemia patients are prone to zinc deficiency [10].

Adding to the nutritional obstacles contributing to zinc deficiency in children of developing countries in general [11, 12], factors contributing to zinc deficiency in thalassemia, in particular, are impaired utilization and excessive losses through ongoing hemolysis and the usage of iron chelators [13–15]. Previous studies reported that deferoxamine and deferiprone might contribute to Zn deficiency in thalassemia, eliminating positive divalent ions, like iron and Zn, into the urine [16, 17].

Few studies reported the effect of zinc deficiency on the endocrine pancreas, but its effect on exocrine pancreatic function had not been widely studied as low levels of zinc in the blood plasma affect the islets of Langerhans secretion and production of insulin. Zinc also plays an important role in forming insulin crystals and the release and transportation of insulin [18]. Moreover, supplementation of zinc to type 2 diabetes patients improved their symptoms of diabetes because it decreases the level of cholesterol and HbA1c levels in the blood [19, 20].

This study aimed to determine the effect of zinc deficiency and iron overload on the endocrine and exocrine function of the pancreas in children with transfusion-dependent thalassemia (TDT) who have secondary hemosiderosis.

## Methods

### Subjects

This cross-sectional study was carried out at the pediatric department, Faculty of Medicine, Minia University, from January 2017 till December 2019. We included in this study 80 children already diagnosed with transfusion-dependent thalassemia (TDT). They were recruited from the Pediatric hematology outpatient clinic and inpatient unit.

Included children were on regular monthly or bi-monthly blood transfusion programs (transfusion-dependent). Their age was ≥ 5 years.

Children with a history of chronic illness other than thalassemia or a change in chelation therapy drug in the last 6 months before participating in the study were excluded.

### Baseline clinical assessment

All included children were subjected to detailed medical history taking and thorough clinical examination with special emphasis on history of the age of the first transfusion, transfusion burden/year (ml/kg/year), history of splenectomy, the average frequency of transfusion, and type and duration of chelation therapy.

### Laboratory analysis

The following laboratory investigations were done for all participants: serum ferritin (mean serum ferritin for each case was calculated from serum ferritin at the time of research and those of the previous year from records), serum zinc (Zn), endocrine pancreatic function (Oral glucose tolerance test (OGTT), fasting insulin level and from them, homeostatic model assessment of insulin resistance (HOMA-IR) was calculated) and exocrine pancreatic function (serum lipase and serum amylase).

From each participant child, we collected 5 ml of venous blood samples and put them on serum separator gel tubes. They were allowed to clot for 30 min at 37 °C before centrifugation for 15 min at 3500 rpm. The expressed serum was used for measurement of serum ferritin and remaining serum was stored at − 20 °C for the other investigations.

Serum zinc was assayed by the colorimetric method (Greiner Diagnostic GmbH, Germany). A normal zinc level range was considered between 63.8–110 μg\dl. Both serum lipase and serum amylase were measured by enzymatic colorimetric assay according to the IFCC-method (Biomed diagnostic, EGY- CHEM for lab technology, for both parameters). Reference values of lipase at 37 °C was ≤38 u\L, while the reference value for serum amylase at 37 °C was between 53 and 123 IU\ml. Serum insulin was assayed by Insulin Human EIA Kit, abcam, ab100578. Fasting insulin levels below ten μIU/L were considered normal [21].

For OGTT, Blood samples for blood glucose were measured at fasting and every half an hour for 2 h (five samples were obtained). Results of the OGTT was graded on NIH criteria: a 2-h glucose level below 140 was considered normal; between 140 and 200, impaired glucose tolerance; and greater than 200, diabetes mellitus [22]. HOMA-IR was calculated according to the following equation: HOMA-IR = fasting insulin **uIU/mL** X fasting glucose (mg/dl) \ 405. HOMA-IR above 1.9 indicated early insulin resistance [23].

The enrolled TDT children were then grouped into two groups; first according to their serum zinc level into low serum zinc group (serum zinc < 63.8 μg\dl) and normal serum zinc group (serum zinc ≥63.8 μg\dl). Then, we grouped the children according to their serum ferritin level into the first group with serum ferritin ≥2500 ng/ml and the second with serum ferritin < 2500 ng/ml.

### Statistical analysis

Data were analyzed using SPSS (statistical package for the social science) version 21 (SPSS Inc., Chicago, Illinois, USA). Quantitative variables were described as mean and standard deviation (SD). Qualitative data were expressed as frequency and percentage.

For the comparison of means, unpaired independent sample student t-test was used. For the comparison of qualitative variables, chi-square test was used. Correlation between two quantitative variables was done by using Pearson’s correlation coefficient. Simple logistic and multivariate regression analyses were done for abnormal serum zinc and serum ferritin levels to determine their odds ratios for exocrine and endocrine pancreatic dysfunction. *p*-value of < 0.05 was considered significant.

## Results

The demographic and clinical data of all the studied TDT children are represented in Table 1. Forty-four TDT children had a subnormal zinc level, they were 21 (47.7%) males and 23 (52.3%) females, their mean age was 11.7 ± 3.7 years. While 36 TDT children had a normal serum Zn level, 19 (52.8%) were males, and 17 (47.2%) were females, with a mean age of 10.7 ± 2.7 years. There were no statistically significant differences between the two groups regarding age and sex.

**Table 1:** Demographic and clinical data of the studied TDT children

Variable	***N*** = 80
**Age of start transfusion (years): Mean ± SD**	2.2 ± 2.7
**Duration of transfusion (years): Mean ± SD**	9.3 ± 5.02
**BMI:** Mean ± SD	16.6 ± 3.02
**Iron chelation therapy:**	
Deferoxamine: n (%)	23 (28.75%)
Deferasirox: n (%)	23 (28.75%)
Deferiprone: n (%)	9 (11.25%)
Combined: n (%)	25 (31.25%)
**Splenectomy:** n (%)	18 (45%)
**Hepatomegaly:**: n (%)	49 (61.25%)

On performing OGTT, TDT children with low serum zinc had significantly more frequently impaired glucose tolerance tests than cases with normal serum zinc, with an odds ratio of 6.4 (95% CI: 1.3–30.7). Also, they had significantly more frequently abnormally high HOMA-IR than cases with normal serum zinc. The odds ratio of high HOMA-IR was 4.2 (95% CI: 1.08–16.6).

Regarding pancreatic exocrine function, serum lipase was significantly higher in TDT children with low serum zinc than those with normal serum zinc. Also, TDT children with low serum zinc had significantly more frequently abnormally high serum lipase than TDT children with normal serum zinc, with an odds ratio of 9.6 (95% CI: 2.9–31.8) (Table 2).

**Table 2:** Endocrine and exocrine pancreatic functions in TDT children according to their zinc status

Variables	Low serum zinc ***N*** = 44	Normal serum zinc ***N*** = 36	p	Odds ratio (95% CI)
**I. Endocrine pancreatic function**				
**1) OGTT**				
Impaired: n (%)	12 (27.3%)	2 (5.6%)	0.01*	6.4 (1.3–30.7)
**2) Insulin (μIu/ml):**				
Mean ± SD	15.4 ± 0.7	12.6 ± 5.8	0.4	
High: n (%)	32 (72.7%)	16 (44.4%)	0.07	0.88 (0.7–1.04)
**3) HOMA-IR**				
Mean ± SD	3.32 ± 3.37	2.7 ± 1.55	0.4	
High: n (%)	24 (54.5%)	8 (22.2%)	0.03*	4.2 (1.08–16.6)
**II. Exocrine pancreatic function**				
**1) Serum amylase (Iu/ml):**				
Mean ± SD	61.95 ± 16.69	61.13 ± 20.8	0.8	
Low: n (%)	14 (31.8%)	12 (33.3%)	0.1	0.79 (0.21–2.9)
**2) Serum lipase (u/l):**				
Mean ± SD	40.23 ± 9.91	31.75 ± 4.9	< 0.001*	
High: n (%)	24 (54.5%)	4 (11.1%)	< 0.001*	9.6 (2.9–31.8)

*CI* confidence interval; *OGTT* Oral glucose tolerance test; *HOMA-IR* Homeostatic model assessment of insulin resistance

* Statistical significance< 0.05

**Fig. 1:**
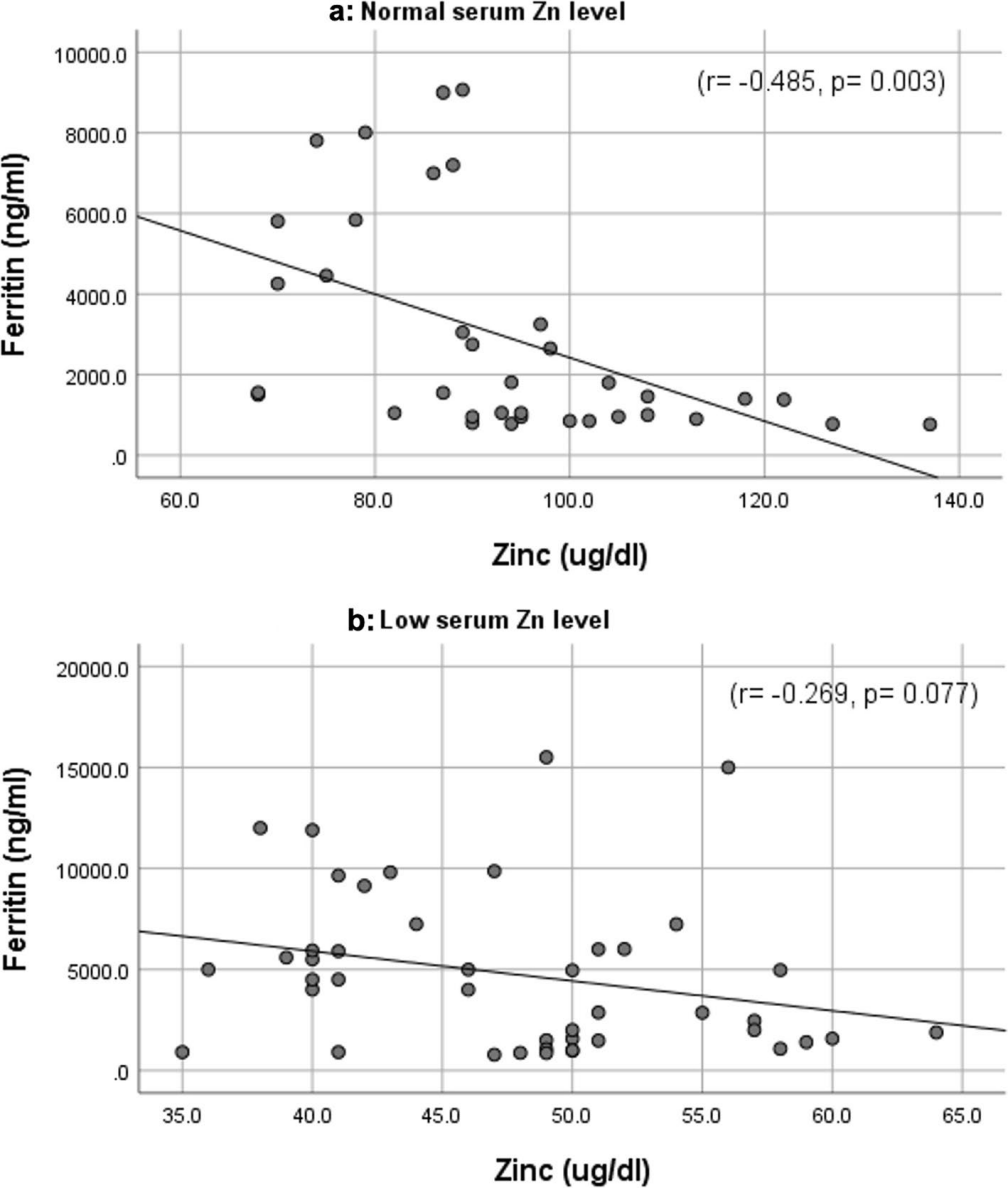
Scatter plots showing correlations between serum zinc and serum ferritin: **a** among TDT children with normal serum zinc and **b** among TDT children with low serum zinc

When we compared serum ferritin of the two groups, we found that TDT children with low zinc had significantly higher serum ferritin (4780.36 ± 3974.02 ng/ml) than TDT children with normal serum zinc level (2895.44 ± 2739.65 ng/ml), as *p* = 0.008. We demonstrated the correlations between serum zinc and serum ferritin in the two groups (Fig. 1).

Moreover, 29 (65.9%) of the TDT children with low zinc had serum ferritin above the 2500 ng/ml threshold. This was significantly more frequent than children with normal serum zinc, as only 14 (38.9%) of them had serum ferritin above the threshold.

Serum zinc was significantly lower in TDT children with serum ferritin above the ferritin threshold than those with serum ferritin below (59.1 ± 20.2 vs. 77.5 ± 28.13), as *p* = 0.02.

When we compared the pancreatic functions of the TDT children according to their ferritin status i.e., having serum ferritin above or below ferritin threshold, which is equal to 2500 ng/ml. TDT children, having a serum ferritin ≥2500 ng/ml, had significantly more frequently impaired oral glucose tolerance test than TDT children below the ferritin threshold, with an odds ratio of 1.4 (95% CI: 0.4–4.5). Also, they had significantly more frequently abnormally high fasting insulin levels than TDT children below the ferritin threshold, with an odds ratio of 4.2 (95% CI: 1.08–16.6). Regarding pancreatic exocrine function, serum lipase was significantly more frequently abnormally high in TDT children having serum ferritin above ferritin threshold than those with serum ferritin below ferritin threshold, with an odds ratio of 4 (95% CI: 1.5–10.8). (Table 3).

**Table 3:** Pancreatic functions and zinc level of TDT children according to their ferritin threshold

Variables	Serum ferritin < 2500 (ng/ml) ***N*** = 40	Serum ferritin ≥ 2500 (ng/ml) N = 40	p	Odds ratio (95% CI)
**I. Endocrine pancreatic function**				
**1) OGTT**				
Impaired: n (%)	6 (15%)	8 (20%)	0.02*	1.4 (0.4–4.5)
**2) Insulin (μIu/ml):**				
Mean ± SD	12.9 ± 10.9	14.35 ± 10.68	0.6	
High: n (%)	16 (40%)	32 (80%)	0.001*	4.2 (1.08–16.6)
**3) HOMA-IR**				
Mean ± SD	2.71 ± 2.29	3.1 ± 2.58	0.6	
High: n (%)	18 (45%)	14 (35%)	0.1	0.7 (0.3–1.6)
**II. Exocrine pancreatic function**				
**1) Serum amylase (Iu/ml):**				
Mean ± SD	57.55 ± 16.97	64.85 ± 18.81	0.2	
Low: n (%)	14 (35%)	12 (30%)	0.07	0.8 (0.3–2)
**2) Serum lipase (u/l):**				
Mean ± SD	40.23 ± 9.91	38.6 ± 8.5	0.03*	
High: n (%)	8 (20%)	20 (50%)	0.005*	4 (1.5–10.8)

*CI* confidence interval; *OGTT* Oral glucose tolerance test; *HOMA-IR* Homeostatic model assessment of insulin resistance

* Statistical significance< 0.05

Pearson’s correlation revealed that serum zinc had significant negative correlations with both serum lipase (r = −0.48, *p* = 0.2) and serum ferritin (r = − 0.38, *p* = 0.01). Serum ferritin had significant positive correlations with OGTT (*r* = 0.31, *p* = 0.005), fasting insulin level (r = 0.39, *p* = 0.005), HOMA-IR (r = 0.43, *p* = 0.006), and serum lipase (*r* = 0.33, *p* = 0.003).

Multiple regression analysis was done to study the effect of having abnormal serum zinc and serum ferritin level as a risk factor for exocrine and endocrine pancreatic dysfunction. Regarding the exocrine pancreatic function expressed by serum amylase and serum lipase. Patients with low zinc levels had the odds of 23.3 times to have high serum lipase more than those with normal serum zinc levels. Also, patients with high serum ferritin levels had the odds of 3.6 times to have high serum lipase, but this did not reach statistical significance in this study. There were no significant effects of serum zinc nor serum ferritin on serum amylase. (Table 4).

**Table 4:** Multivariate regression analysis of the effect of abnormal serum zinc and serum ferritin on exocrine and endocrine pancreatic function in TDT children

		Odds ratio	95% CI of OR		p
			Lower	Upper	
**Exocrine function**					
**- Amylase**	**Zinc**	0.605	0.15	2.3	0.4
	**Ferritin**	0.879	0.22	3.4	0.8
**- Lipase**	**Zinc**	23.378	2.50	218.4	0.006*
	**Ferritin**	3.651	0.69	19.1	0.1
**Endocrine function**					
**- OGTT**	**Zinc**	6.310	0.66	59.8	0.1
	**Ferritin**	1.053	0.18	5.9	0.9
**- HOMAIR**	**Zinc**	3.9	0.99	15.7	0.04*
	**Ferritin**	1.5	0.38	6.07	0.5
**- Fasting Insulin**	**Zinc**	2.1	0.9	7.3	0.07
	**Ferritin**	1.667	0.087	31.869	0.2

*CI* confidence interval; *OR* Odds ratio; *OGTT* Oral glucose tolerance test; *HOMA-IR* Homeostatic model assessment of insulin resistance.

* Statistical significance< 0.05

For the pancreatic endocrine dysfunction, patients with low serum zinc had the odds of 3.9 times to have an abnormal HOMA-IR more than those with normal serum zinc. Moreover, patients with low serum zinc levels had odds of 6.3 times to have impaired OGTT and odds of 2.1 times to have high fasting insulin, but both odds ratios were not statistically significant.

Nevertheless, patients with high serum ferritin levels had odds of 1.5 times to have an abnormal HOMA-IR and 1.6 times to have a high fasting insulin level. However, again, these two odds ratios did not reach statistical significance in this study (Table 4).

## Discussion

This study aimed to study the effect of zinc deficiency and iron overload on the pancreatic functions in children with transfusion-dependent thalassemia.

We compared the endocrine pancreatic functions in TDT children according to their zinc level; we found that impaired glucose tolerance tests and increased insulin resistance were more frequent in TDT children with zinc deficiency. Many researchers studied the relationship between glucose metabolism and zinc level in thalassemia; they showed that impaired glucose metabolism and low serum zinc levels were common among patients [24, 25]. Furthermore, another study on patients with thalassemia showed that a decline in serum zinc was associated with a consistent impairment in glucose tolerance test [14].

Zinc deficiency, in general, was linked to insulin resistance. An Australian study on adults with prediabetes showed that higher zinc levels were associated with decreased insulin resistance [26], and serum zinc was negatively associated with insulin resistance in another study on non-diabetics [27]. Adding to the previous studies, cross-sectional analyses reported that children with lower serum zinc concentrations and low dietary zinc intakes have significantly higher serum insulin concentrations and insulin resistance indexes [28–30].

A recent study by Ravi Kant et al. demonstrated that increasing zinc levels improves pancreatic function in normoglycemic adults and decreases insulin resistance in prediabetic adults. Since zinc has been shown to play an essential role in both insulin secretion and insulin action, it is not surprising that zinc levels affect both pancreatic function and insulin resistance [31]. These data agree with the recommendations of De Sanctis and his study group in 2016. They recommended serum zinc levels to be monitored in patients with thalassemia major, as it provides valuable complementary information regarding glucose metabolism [6].

Regarding pancreatic exocrine function, zinc-deficient TDT children had higher mean serum lipase and had more frequently an abnormally high serum lipase (> 38 U/L) than TDT children with normal serum zinc. The relation of zinc deficiency with exocrine pancreatic dysfunction may be less clear than its relationship with endocrine pancreatic function, as there is no clear data on whether zinc deficiency aggravates or results from chronic pancreatitis. On the one hand, micro-and macronutrients and several other nutritional deficiencies were found in subjects with chronic pancreatitis, including lipid-soluble vitamins A, D, E, and K, zinc, and others [32]. Moreover, in patients with chronic pancreatitis, erythrocyte and serum zinc levels were reported to be significantly lower [33, 34]. On the other hand, zinc deficiency may modulate pancreatic digestive enzyme activity by decreasing transporter protein ZnT2 expression. This transporter protein has been implicated in zinc transport into zymogen granules of the exocrine pancreas for the metallation of digestive proenzymes [35, 36]. Additionally, experimental studies strongly correlated zinc deficiency with pancreatic function and structure, as rats’ long-term feeding a zinc-deficient diet resulted in pancreatic acinar cell degeneration [37, 38].

TDT children with low serum zinc had significantly higher serum ferritin than those with normal serum zinc. Iron overload leads to overproduction of free radicles aggravating oxidative stress, which alters the levels of antioxidant enzymes in serum, causing zinc and other trace elements deficiency [39]. Additionally, increased iron can inhibit zinc absorption in the gastrointestinal tract because iron and zinc compete for the transferrin binding sites in blood and inhibit each other absorption —moreover, iron chelators in patients with thalassemia chelates zinc and other essential minerals besides iron [15].

This study found serum zinc to have significant negative correlations with both serum lipase and serum ferritin. These correlations go with several studies that found ferritin levels to have significant negative correlations with plasma zinc levels [40–43], which supports that iron overload aggravates zinc deficiency [39]. The negative association of serum zinc with serum lipase confirms that zinc deficiency and pancreatitis have a reciprocal relationship [33, 34].

Previous studies had set serum ferritin ≥2500 ng/ml as a threshold above which cardiac, and many iron overload-related organ dysfunctions are aggravated. Serum ferritin can reliably predict cardiac siderosis and endocrine disease in thalassemia when equal to or above 2500 ng/ml [44].

When we compared pancreatic functions of TDT children according to their serum ferritin level, cases with serum ferritin above the ferritin threshold had higher first-hour blood glucose and had more frequently impaired OGTT and high fasting insulin levels than cases with a serum ferritin below ferritin threshold. Excessive iron deposition in the pancreas leads to abnormal glucose metabolism [45, 46]. as iron overload-generated oxidative damage and functional impairment of insulin-producing pancreatic ß-cells lead to glucose dysregulation [47]. Besides, insulin resistance developed from iron overload-induced hepatic dysfunction [48].

Regarding pancreatic exocrine function relation to serum ferritin level, TDT children with serum ferritin ≥2500 ng/ml had significantly higher serum lipase (> 38 U/L) than TDT children having serum ferritin below this level. ***Andersson*** and co-workers suggested that secondary hemochromatosis is one of the toxic factors causing chronic pancreatitis in thalassemia [49]. They attributed that chronic oxidative stress induced by hemochromatosis is toxic to the pancreatic cells [50]. Other studies validated the direct link of iron overload to exocrine and endocrine pancreatic dysfunction through quantitative measuring the pancreatic iron by MRI [51–53].

In this study, zinc deficiency is a significant risk factor for exocrine and endocrine pancreatic dysfunction as zinc is involved in many of these processes within the pancreas, including glucagon secretion, digestive enzyme activity, and insulin packaging, secretion, and signaling. So, zinc deficiency impairs many vital processes of the pancreas leading to exocrine dysfunction and impairment of systemic glycemic control [36], aggravating the iron overload-induced pancreatic injury.

## Conclusion

Zinc deficiency aggravates iron-induced pancreatic exocrine and endocrine dysfunction in children with transfusion-dependent thalassemia. Therefore, we recommend regular serum zinc level monitoring and zinc supplementation in children with TDT, together with robust iron chelation therapy, as this may slow the progression of pancreatic function deterioration in those children. However, further studies are needed to study the effects of zinc supplementation on pancreatic function in TDT children.

## Data Availability

The datasets analyzed during the current study are available from the corresponding author on reasonable request.

## Abbreviations

TDT: Transfusion-dependent thalassemia
OGTT: Oral glucose tolerance test
HOMA-IR: Homeostatic model assessment of insulin resistance
Zn: Zinc
